# Abdominal Apoplexy: Sudden Death Due to Massive, Nontraumatic Intra-abdominal Hemorrhage

**DOI:** 10.7759/cureus.47335

**Published:** 2023-10-19

**Authors:** Biliana Mileva, Mihaela Georgieva, Ivan I Tsranchev, Metodi Goshev, Milena Gulinac, Alexandar Alexandrov

**Affiliations:** 1 Department of Forensic Medicine and Deontology, Medical University Sofia, Sofia, BGR; 2 Department of Forensic Medicine and Deontology, Medical University of Plovdiv, Plovdiv, BGR; 3 Department of General and Clinical Pathology, Medical University of Plovdiv, Plovdiv, BGR

**Keywords:** sudden death, forensic pathology, idiopathic spontaneous intraperitoneal hemorrhage, intraperitoneal hemorrhage, abdominal apoplexy

## Abstract

Abdominal apoplexy or idiopathic spontaneous intraperitoneal hemorrhage (ISIH), the modern way to describe this condition, is a rare phenomenon representing intra-abdominal bleeding with no traumatic origin. The condition is severe and potentially life-threatening. A man in his 70s was found dead at his home. The autopsy revealed a massive hemoperitoneum with no visible traumatic injuries externally or internally. The source of the bleeding was unknown despite the careful examination of the internal organs and splanchnic vessels. The cause of death was attributed to massive exsanguination due to the rupture of a small blood vessel as a complication of generalized arteriosclerosis and hypertensive disease. Despite its rare occurrence, the forensic pathologist should always consider this pathological condition when, during the autopsy, a massive intraabdominal hemorrhage with or without a visible source of the bleeding is discovered in the absence of any visible traumas.

## Introduction

The term “apoplexy” originates from the Greek word “apoplexia” or “apoplessein,” which means sudden pаralysis from breаking or obstruction of a blood vessel in the brain or to strike down and incapacitate, which was first used in association with “cerebral apoplexy” [1-3]. Abdominal apoplexy is the ancient, historical term for intraperitoneal hemorrhage due to the rupture of small intraperitoneal or extraperitoneal abdominal blood vessels in the absence of trauma or underlying pathology [2,4,5]. Nowadays, this condition is called idiopathic spontaneous intraperitoneal hemorrhage (ISIH) and is extremely rare, with potentially fatal outcomes [1,4,6,7].

## Case presentation

A male in his 70s was found unresponsive on the floor of his home. There was no evidence of forced entry into the apartment, but still, a medicolegal autopsy was ordered by the police to determine the cause and manner of death.

**Figure 1:**
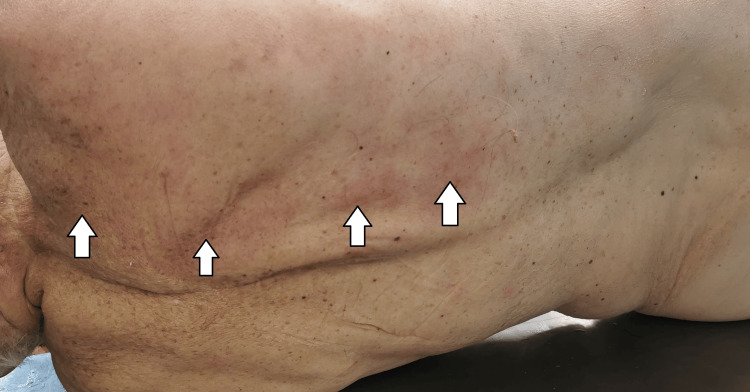
Pale and not well presented postmortem lividity (livor mortis)

The deceased's corpse was found on the floor in the corridor next to his room, lying on his right side. During the external examination of the body, no traumatic injuries were found. The lividities were scanty, present on the right side of his body, and fixed (Figure 1).

**Figure 2:**
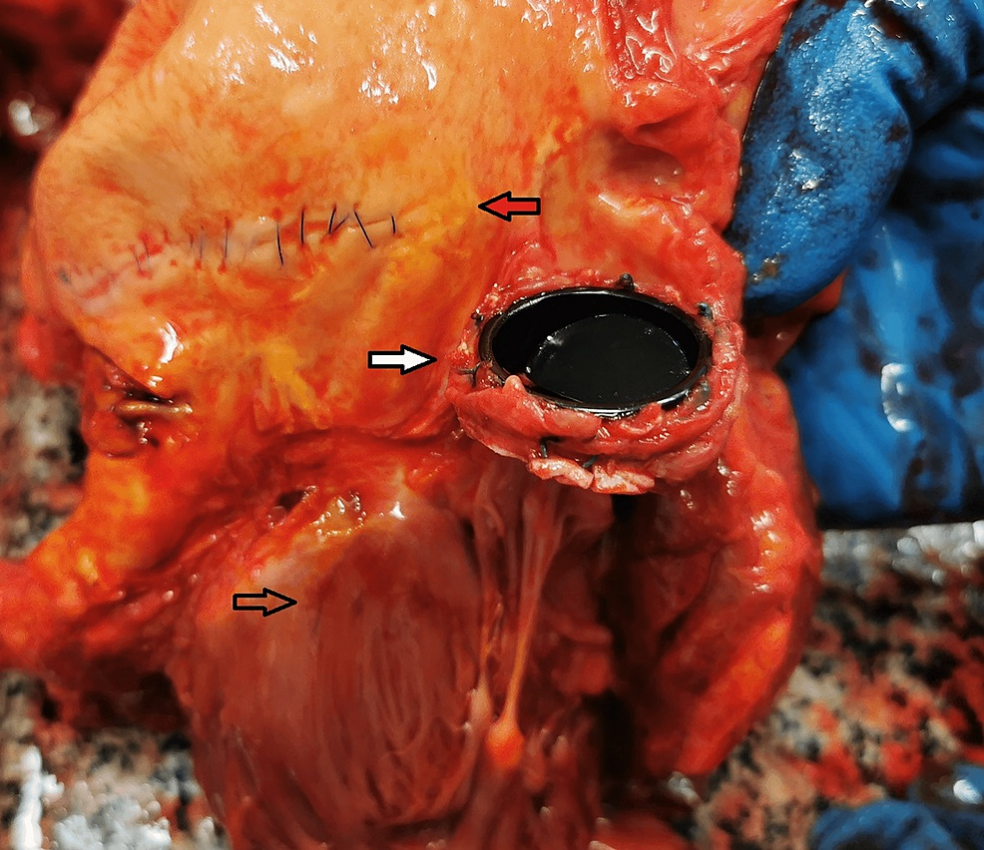
Subendocardial hemorrhages – spots of Minakov (transparent arrow); aortic valve replacement (white arrow); atherosclerotic changes of the aorta (red arrow).

**Figure 3:**
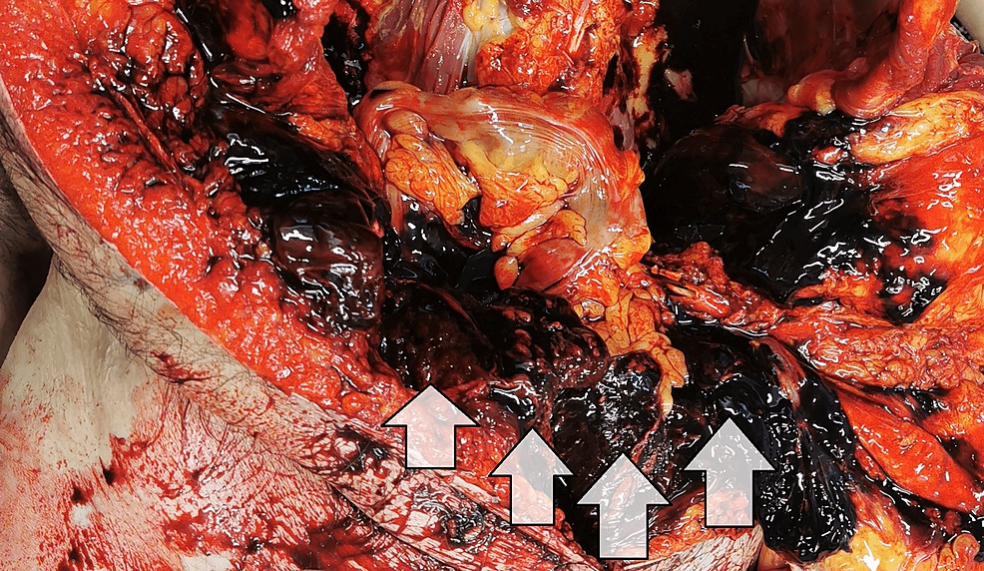
Accumulation of blood and blood clots inside the abdominopelvic cavity

The muscle stiffening was well-developed. No traumatic injuries were present on the body. The internal examination showed the following findings: generalized complicated atherosclerosis with primary affection of the abdominal aorta and its branches; signs of hypertensive and chronic ischemic heart disease; replacement of the aortic valve (Figure 2); massive accumulation of blood in the abdominopelvic cavity (approximately 2.5-3 L of dark liquid and clotted blood), with infiltration of the fat tissue which surrounds the internal organs (Figure 3).

The blood and the clots were evacuated, and all of the internal organs were carefully examined in situ (the spleen, liver, bowel, kidneys, mesentery, prostatic gland, and urinary bladder), and no source of bleeding was found. The splanchnic vessels were without any significant deviation. The internal organs were pale, and subendocardial hemorrhages were present - spots of Minakov - signs of blood loss (Figure 2). Additional careful examination of the body did not reveal any visible traumas. The histopathologic evaluation of the tissue samples showed morphologic signs of acute hemorrhagic shock and confirmed the macroscopic findings. The results from the toxicology report did not show evidence of intoxication. Based on the thorough examination of the deceased body and the results from the additional examinations, the cause of death was attributed to a massive exsanguination due to a spontaneous intraabdominal hemorrhage from the rupture of an unidentified small blood vessel, not caused by a traumatic event. The postmortem interval was estimated to be between the end of the first and the beginning of the second day.

## Discussion

The first described case associated with idiopathic spontaneous intraperitoneal hemorrhage was in 1909 by Maurice Barber in a woman two days after giving birth [8]. He concluded that "the intra-abdominal hemorrhage which occurred in the following case must, I think, be a very unusual complication of labor." [8]. Later, in 1931, the term "abdominal apoplexy" was coined by Green and Powers as a comparison to its cerebral counterpart [4,7,9,10]. Nowadays, abdominal apoplexy refers to the spontaneous rupture of an abdominal blood vessel after excluding hemorrhage from gross aneurysm, gynecological lesions, visceral malignancies, trauma or any other prominent pathological states [2,11]. Commonly, the rupture of the blood vessel occurs at the site of an aneurysm. The pathophysiology of the process is explained by the weakness of the tunica media in a small vessel with angiopathy, predisposing to rupture when there is an abrupt increase in blood pressure [12,13]. Such aneurysms are typically located at secondary or tertiary branches of the aorta; 60% involve splenic artery, 22% renal, 10-20% hepatic, 5.5% superior mesenteric artery, 4% celiac, gastrointestinal/epiploic arteries, 3% intestinal artery, and 1.5% duodenal/pancreatic arteries [1,5,6,12]. Wang and Xiu report a case of abdominal apoplexy with two bleeding sources - rupture of the gastroduodenal artery and inferior pancreaticoduodenal artery [14]. In his study, DiMaio describes three cases of chronic alcoholics with liver cirrhosis, and all died of massive intra-abdominal hemorrhage not associated with trauma. One died in a hospital, showing evidence of a disseminated intravascular coagulopathy. He explains this coagulopathy with alcoholic liver disease or at least in conjunction with liver cirrhosis [15]. Debouit et al. described a similar case of a 39-year-old woman, a chronic alcoholic with liver cirrhosis, who died from massive abdominal bleeding from an unknown source [1]. Despite the careful inspection of the abdominal cavity, pelvic area, and retroperitoneal compartment, the bleeding source is unknown in nearly 30% of cases [5,7,16].

The idiopathic spontaneous intraperitoneal hemorrhage is rarely observed in medico-legal practice. It is a diagnosis of exclusion, so a thorough examination is needed since the location of a small vessel rupture in a large bloody field might be difficult and sometimes impossible [17]. The most important in such cases from a forensic point of view is to exclude a possible trauma that led to the abdominal bleeding and the person's death.

## Conclusions

We present a peculiar case of an abdominal apoplexy with an unidentified bleeding site. The analyses of the autopsy findings led us to conclude that the described condition resulted from a small vessel rupture associated with generalized complicated arteriosclerosis and hypertensive disease.
